# Tunable Nanosensor Based on Fano Resonances Created by Changing the Deviation Angle of the Metal Core in a Plasmonic Cavity

**DOI:** 10.3390/s18041026

**Published:** 2018-03-29

**Authors:** Qiong Wang, Zhengbiao Ouyang, Yiling Sun, Mi Lin, Qiang Liu, Guoliang Zheng, Junxing Fan

**Affiliations:** 1 THz Technical Research Center of Shenzhen University, Shenzhen University, Shenzhen 518060, China; qwang@szu.edu.cn (Q.W.); sunyl@szu.edu.cn (Y.S.); linfengas111@szu.edu.cn (M.L.); qliu@szu.edu.cn (Q.L.) zhgl@szu.edu.cn (G.Z.); fanjunxingfjx@yeah.net (J.F.); 2 College of Electronic Science &Technology, Shenzhen University, Shenzhen 518060, China

**Keywords:** tunable nanosensor, Fano resonances, plasmonic cavity, finite element method

## Abstract

In this paper, a type of tunable plasmonic refractive index nanosensor based on Fano resonance is proposed and investigated. The sensor comprises a metal-insulator-metal (MIM) nanocavity with a center-deviated metal core and two side-coupled waveguides. By carefully adjusting the deviation angle and distance of the metal core in the cavity, Fano resonances can be obtained and modulated. The Fano resonances can be considered as results induced by the symmetry-breaking or geometric effect that affects the field distribution intensity at the coupling region between the right waveguide and the cavity. Such a field-distribution pattern change can be regarded as being caused by the interference between the waveguide modes and the cavity modes. The investigations demonstrate that the spectral positions and modulation depths of Fano resonances are highly sensitive to the deviation parameters. Furthermore, the figure of merit (FOM) value is calculated for different deviation angle. The result shows that this kind of tunable sensor has compact structure, high transmission, sharp Fano lineshape, and high sensitivity to the change in background refractive index. This work provides an effective method for flexibly tuning Fano resonance, which has wide applications in designing on-chip plasmonic nanosensors or other relevant devices, such as information modulators, optical filters, and ultra-fast switches.

## 1. Introduction

In the past decade, the surface plasmon resonances of noble metal nanostructures have attracted considerable research interest due to their unusual abilities to manipulate light within subwavelength dimensions and to produce extremely strong local electromagnetic fields near the metal surfaces [1,2,3]. As we know, the plasmonic structures described by quasi-lorentzian profiles usually exhibit a symmetric and wide lineshape in transmission spectra [4,5,6]. This would limit their practical applications. Distinctively different from quasi-lorentzian resonances, Fano resonance generally has an asymmetric and sharp lineshape [7,8,9]. More importantly, it is very sensitive to the changes in geometry and local dielectric environment, which can lead to many potential applications in bio/chemical sensors, information modulators, optical filters, ultra-fast switches, and so on [10,11,12,13].

Fano resonance has been known for many years in quantum systems [14,15]. Recently, it has been applied in other fields, such as nanostructures based on optical cavities, waveguide arrays, or nanoparticle clusters [16,17,18,19]. Specifically, rich Fano lineshapes can be obtained in plasmonic systems. Research on the tunability of plasmonic Fano resonances has attracted increasing interest, since it exhibits great flexibility for designing various devices [20,21,22,23,24,25,26,27,28]. For example, it has been reported that Fano resonance can be easily adjusted in a plasmonic Fabry-Perot cavity with a baffle, which is useful for designing wavelength division multiplexers [24]. Moreover, a plasmonic slow-light sensor can be realized in double stacked cavities, in which the Fano resonance can be modulated by changing the degree of asymmetry of the cavities [27]. Additionally, a kind of electrically tunable Fano-type resonance of asymmetric metal wire pairs has been achieved by controlling the varactor diode loaded on the plasmonic nanostructure [28].

As we know, the most efficient way to induce Fano resonance in a plasmonic system is to introduce symmetry breaking into structure, such as the Fano resonances produced in H-shaped, E-shaped, disk-ring, and cascading-cavity nanostructures [29,30,31,32,33]. Symmetry breaking is always realized by making a deviation of a part of structure along a single direction. Little research has been conducted on studying the effects of deviation along different directions. Therefore, in this paper, a kind of simple nanostructure with symmetry breaking along a wide range of directional changes is considered to adjust Fano resonances. This may result in more flexibility for adjusting Fano resonances. The proposed structure consists of a metal-dielectric-metal nanocavity with a center-deviated metal core and two side-coupled waveguides. By changing the deviation angle of the metal core along different directions, and adjusting the corresponding deviation distance for each deviation angle in the cavity, Fano resonances can be obtained and tuned. Furthermore, the sensitivity to the background refractive index is investigated for different cases of deviation. The figure of merit (FOM) values are also calculated and compared. This kind of sensor has a compact structure that is appropriate for integration with other on-chip devices, which plays an important role in nano-integrated plasmonic systems.

## 2. Structure Model and Design

**Figure 1:**
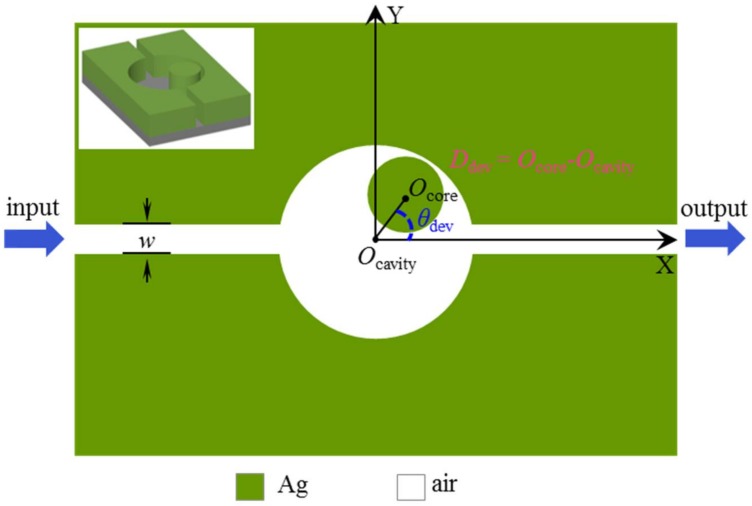
Schematic illustration of a plasmonic MIM nanostructure consisting of a core-deviated cavity and two side-coupled waveguides, where the core is deviated from the center of the cavity with a distance *D*_dev_ and a direction angle *θ*_dev_.

Figure 1 shows the X-Y cross-section of the nanoscale plasmonic MIM system that is studied in this paper. The left inset in Figure 1 is the three-dimensional structure. When the structure is tall enough along the z-axis direction (the height of the structure can be set as 250 nm, referring to a simple example [34]), it can be regarded as a two-dimensional one. Similar results can be obtained, and the complexity of calculation is reduced greatly. Therefore, we consider a two-dimensional model for the demonstration of principles. It is necessary to emphasize that the MIM structure is chosen because it has the advantages of deep-subwavelength field confinement, low bend loss and easy integration [35,36,37]. The green and white areas represent the materials of noble metal and air (ε_air_ = 1.0), respectively. Two waveguides are side-coupled with a circular air cavity, the radius of which is set as R_cavity_ = 260 nm. The cavity consists of a center-deviated metal core with a radius of R_core_ = 90 nm. O_cavity_ and O_core_ denote the cavity and core center points, respectively. In order to introduce symmetry breaking into the nanocavity, the position of the metal core is changed through a wide range. For convenience, the deviation angle and distance are defined as θ_dev_ and D_dev_, where D_dev_ = O_core_ − O_cavity_. The width of waveguides is set as W = 50 nm. The noble metal is chosen as silver, whose frequency-dependent complex relative permittivity is characterized by the Drude model [38,39]:

(1) $$\varepsilon_{m}(\omega )=\varepsilon_{\infty }-\frac{\omega_{p}^{2}}{\omega (\omega +i\gamma )}$$

where *ε*_∞_ is the dielectric constant at the infinite frequency, *γ* is the electron collision frequency, *ω* is the frequency of the incident light and *ω*_p_ is the bulk plasma frequency. The parameters are *ε*_∞_ = 3.7, *ω*_p_ = 1.38 × 10^16^ Hz and *γ* = 2.73 × 10^13^ Hz. 

In order to understand the asymmetric cavity structure, the transmission function can be considered using the transfer matrix method [40,41,42]. The scattering property of the system for incident waves at a frequency *ω* can be described as

(2) $$\left[\begin{array}{c} b_{2}\\ a_{2} \end{array}\right]=T_{c}\left[\begin{array}{c} a_{1}\\ b_{1} \end{array}\right]=\left[\begin{array}{cc} 1-\frac{i\eta }{\omega -\omega_{0}} & \frac{-i\eta }{\omega -\omega_{0}}\\ \frac{i\eta }{\omega -\omega_{0}} & 1+\frac{i\eta }{\omega -\omega_{0}} \end{array}\right]\left[\begin{array}{c} a_{1}\\ b_{1} \end{array}\right]$$

where *ω*_0_ and *η* are the center frequency and the width of the resonance of the cavity with a metal core in the center (*D*_dev_ = 0), respectively. The transfer matrix relates the incoming and outgoing wave amplitudes *a*_1_ and *b*_1_ on the left side of the cavity, to the outgoing and incoming wave amplitudes *b*_2_ and *a*_2_ on the right side. The center-deviated metal core can be considered an element affecting the phase and reflection of the left waves in the cavity, with a transfer matrix determined as

(3) $$T_{l}=\frac{1}{i\sqrt{1-{r_{l}}^{2}}}\left[\begin{array}{cc} -1 & -r_{l}\\ r_{l} & 1 \end{array}\right]\left[\begin{array}{cc} e^{i\varphi_{l}} & 0\\ 0 & e^{-i\varphi_{l}} \end{array}\right]$$

Meanwhile, it also affects those of the right waves in the cavity, with a transfer matrix determined as

(4) $$T_{r}=\frac{1}{i\sqrt{1-{r_{r}}^{2}}}\left[\begin{array}{cc} e^{i\varphi_{r}} & 0\\ 0 & e^{-i\varphi_{r}} \end{array}\right]\left[\begin{array}{cc} -1 & -r_{r}\\ r_{r} & 1 \end{array}\right]$$

Therefore, the transfer matrix *T*_s_ for the entire system is denoted by

(5) $$T_{s}=\frac{-1}{\sqrt{\left(1-{r_{l}}^{2})(1-{r_{r}}^{2}\right)}}\left[\begin{array}{cc} -1 & -r_{l}\\ r_{l} & 1 \end{array}\right]\left[\begin{array}{cc} e^{i\varphi_{l}} & 0\\ 0 & e^{-i\varphi_{l}} \end{array}\right]\left[\begin{array}{cc} 1-\frac{i\eta }{\omega -\omega_{0}} & \frac{-i\eta }{\omega -\omega_{0}}\\ \frac{i\eta }{\omega -\omega_{0}} & 1+\frac{i\eta }{\omega -\omega_{0}} \end{array}\right]\left[\begin{array}{cc} e^{i\varphi_{r}} & 0\\ 0 & e^{-i\varphi_{r}} \end{array}\right]\left[\begin{array}{cc} -1 & -r_{r}\\ r_{r} & 1 \end{array}\right]$$

From Equation (5), the transmission of the entire system *T*(*ω*) can be written out as

(6) $$T(\omega )={\left|\frac{1}{T_{s,22}}\right|}^{2}\;={\left|\frac{-\sqrt{\left(1-r_{l}^{2})(1-r_{r}^{2}\right)}(\omega -\omega_{0})}{\left[e^{-i(\varphi_{l}+\varphi_{r})}-r_{l}r_{r}e^{i(\varphi_{l}+\varphi_{r})}\right](\omega -\omega_{0})+i\eta \left[r_{l}r_{r}e^{i(\varphi_{l}+\varphi_{r})}+e^{-i(\varphi_{l}+\varphi_{r})}-(r_{r}e^{-i(\varphi_{l}-\varphi_{r})}+r_{l}e^{i(\varphi_{l}-\varphi_{r})})\right]}\right|}^{2}$$

When the deviation angle and distance of the metal core are adjusted, the effect of phase and reflection of the metal core to the left- and right-side waves will change, which leads to corresponding changes for the parameters *η*, *r_l_*, *r_r_*, *φ_l_*, *φ_r_*. Thus, a complex response will take place for the transmission *T*(*ω*), resulting in the formation of Fano resonance. In the following section, by using Comsol software (finite element method), the transmission characteristic is numerically simulated to investigate the optical responses of the designed plasmonic system in detail. Perfectly matched layers are added around the calculated domain to absorb the electromagnetic wave going out of the structure. The structure is divided into a grid of about 3.5 × 10^4^ small cells.

## 3. Results and Discussion

In this section, we will investigate the properties of transmission when the metal core is deviated from the center of the cavity, i.e., the deviation distance *D*_dev_ ≠ 0_._ The fundamental TM mode is excited at the input waveguide. *P*_in_ and *P*_out_ describe the incident power and transmitted power, which are detected by two power monitors set at the input and output ports, respectively. The transmission of output waveguide is calculated as *P*_out_/*P*_in_.

**Figure 2:**
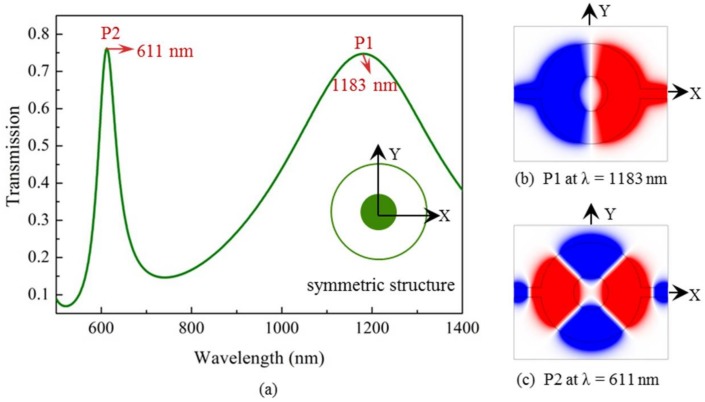
(**a**) Transmission for the case of the symmetric structure with the metal core located at the center of the cavity (*D*_dev_ = 0), and its *H_z_* field distributions at the transmission peaks of (**b**) λ = 1183 nm, P1 and (**c**) λ = 611 nm, P2.

As two special cases, deviations along the vertical and horizontal directions are respectively considered first. For comparison, it is necessary to study the resonance characteristics of the symmetric structure. In this case, the metal core is located at the center of the cavity, i.e., at a deviation distance *D*_dev_ = 0. Figure 2a shows the calculated transmission as a function of wavelength. The inset in Figure 2a shows the symmetric cavity. We can find that two high-transmission peaks (denoted by P1 and P2) appear at 1183 nm and 611 nm. They exhibit a symmetric lorentzian-like lineshape, corresponding to dipolar and quadrupolar modes, respectively, as can be seen from the simulated *H_z_* magnetic field distributions in Figure 2b,c. The red and blue domains denote positive and negative magnetic fields, respectively. It is obvious that both of the modes are symmetric about the *x*-axis.

**Figure 3:**
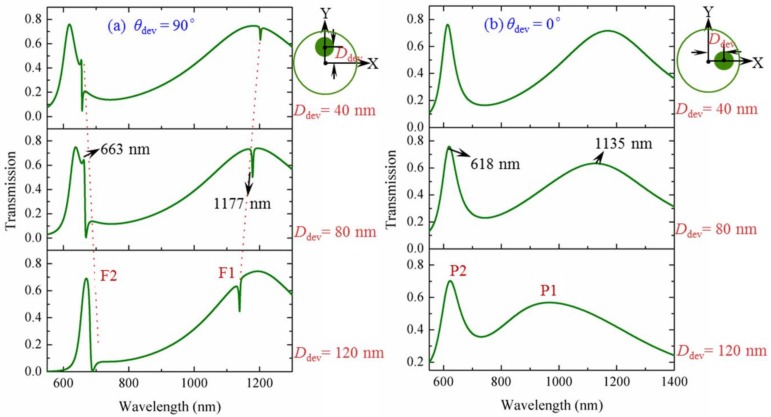
Transmissions for the deviation cases of the metal core moving along (**a**) the vertical direction and (**b**) the horizontal direction with a change of *D*_dev_ = 40 nm, 80 nm and 120 nm.

Figure 3a,b show the calculated transmissions for the cases of vertical (*θ*_dev_ = 90°) and horizontal (*θ*_dev_ = 0°) deviation, respectively. We study the spectral properties with a change in deviation distance *D*_dev_ along the vertical (or horizontal) direction at *D*_dev_ = 40 nm, 80 nm and 120 nm. The position change of the metal core in the cavity is shown on the right side of each figure. For the case of vertical deviation as shown in Figure 3a, there are two evident differences when compared with the spectrum of symmetric structure. A transmission dip appears around the transmission peak P1. Meanwhile, a change from transmission peak to valley is formed around the transmission peak P2. The two changes above can be considered typical Fano resonances (denoted by F1 and F2), which are the results of the symmetry breaking introduced in the cavity. Moreover, the Fano resonances become sharper with the increase of *D*_dev_ due to the increasing asymmetry in the structure. In Figure 3b, we can see that the transmission spectra are different from those in Figure 3a. Although symmetry breaking is introduced into the cavity when the metal core is deviated along the horizontal direction, Fano resonance cannot be obtained; the transmission peaks of P1 and P2 just shift a little.

**Figure 4:**
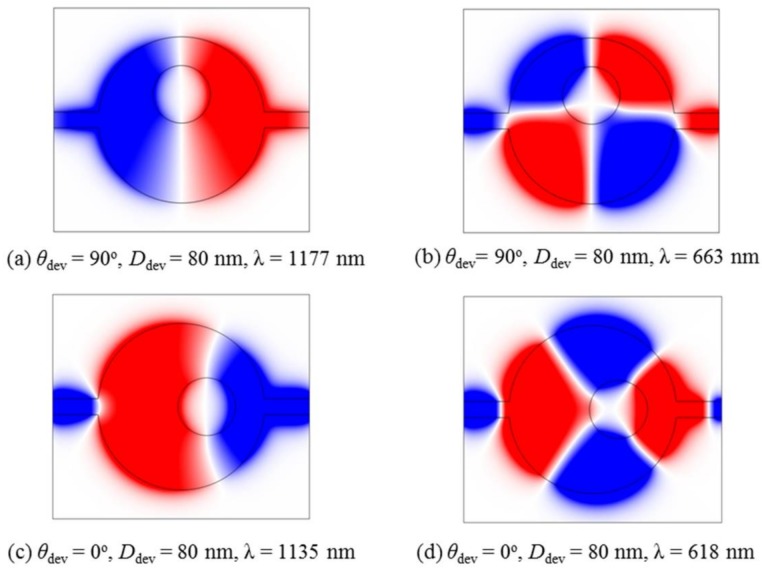
*H_z_* magnetic-field distributions of the vertical deviation of the metal core at the highest transmission of Fano resonances, (**a**) λ = 1177 nm and (**b**) λ = 663 nm; and *H_z_* distributions of the horizontal deviation of the metal core at the transmission peaks of (**c**) λ = 1135 nm and (**d**) λ = 618 nm.

In order to further understand the above phenomenon, the *H_z_* magnetic-field distributions of *D*_dev_ = 80 nm are chosen for both the vertical and horizontal deviations. Figure 4a,b show the case of vertical deviation. The wavelengths are chosen at the highest transmission of each Fano resonance, respectively, i.e., λ = 1177 nm and λ = 663 nm. These correspond to the dipolar and quadrupolar modes, respectively. It can be found that the symmetry of the *H_z_* magnetic-field is destroyed when the metal core moves along the vertical direction in the cavity, which is the main reason for the production of Fano resonances. Figure 4c,d show the case of horizontal deviation. The wavelengths are chosen at λ = 1135 nm and λ = 618 nm, which correspond to the two transmission peaks. The *H_z_* magnetic-field distributions still retain symmetry about the x-axis, although the metal core moves along the horizontal direction in the cavity. As a result, Fano resonance cannot be obtained. This indicates that the formation of Fano resonance is closely related to the asymmetry of field distribution, which can be realized by reducing the symmetry of the structure.

**Figure 5:**
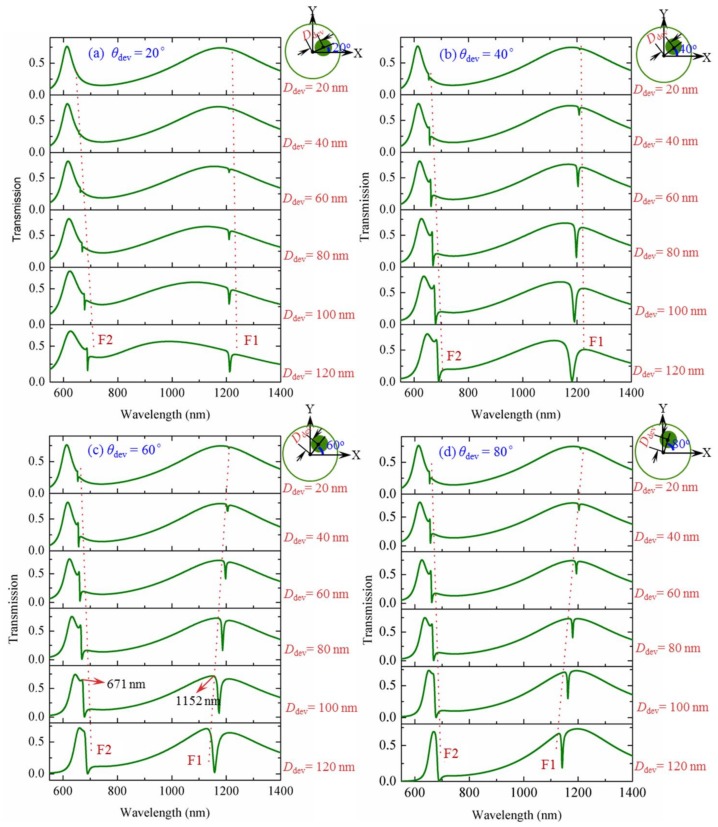
Transmissions for the deviation cases of the metal core moving along different vertical direction (**a**) *θ*_dev_ = 20°; (**b**) *θ*_dev_ = 40°; (**c**) *θ*_dev_ = 60° and (**d**) *θ*_dev_ = 80°. The deviation distance is set as *D*_dev_ = 20 nm, 40 nm, 60 nm, 80 nm, 100 nm and 120 nm for each case of *θ*_dev_.

Next, we will investigate the change of Fano resonances when the deviation angle *θ*_dev_ is adjusted across a wide range. Figure 5 shows the transmissions for the cases of *θ*_dev_ = 20°, 40°, 60° and 80°. The deviation distance is calculated at the values of *D*_dev_ = 20 nm, 40 nm, 60 nm, 80 nm, 100 nm and 120 nm for each deviation angle. For a fixed *θ*_dev_ (0° < *θ*_dev_ < 90°), when *D*_dev_ is increased from a small value, two Fano resonances start to appear (denoted by F1 and F2) around the transmission peaks P1 and P2, respectively. The spectral positions and modulation depths of Fano resonances are highly sensitive to the deviation parameters. The Fano resonance wavelength shifts a little with the increase of *D*_dev_. More importantly, Fano resonance shape becomes sharper with increasing *D*_dev_. This is due to the increasing asymmetry in the structure.

**Figure 6:**
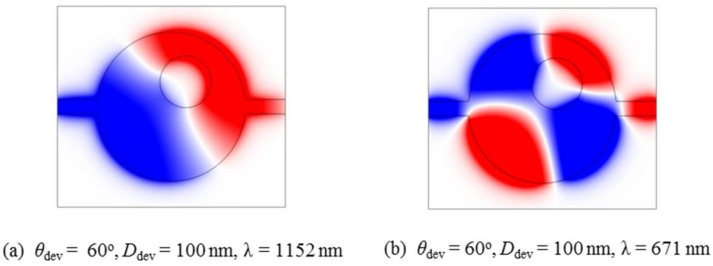
*H_z_* magnetic field distributions for the parameters of *θ*_dev_ = 60° and *D*_dev_ = 100 nm are simulated. The wavelengths are chosen as (**a**) λ = 1152 nm and (**b**) λ = 671 nm.

**Figure 7:**
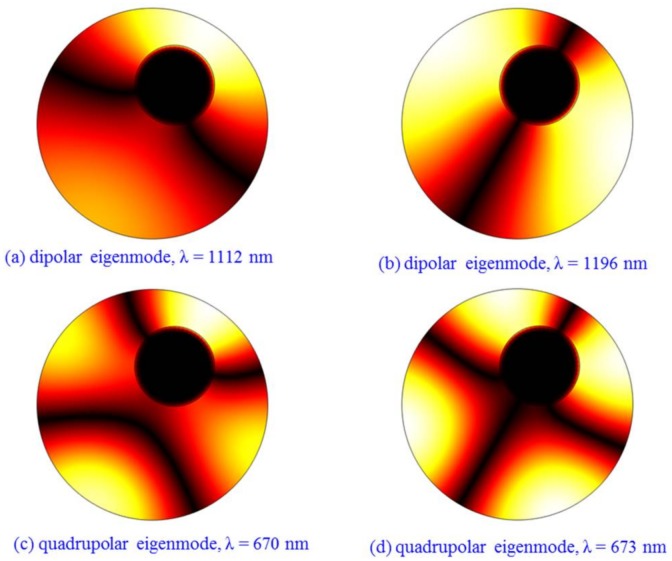
The eigenmodes of |*H*| distributions of a circular cavity with a center-deviated metal core (*θ*_dev_ = 60^o^ and *D*_dev_ = 100 nm). The wavelengths are at (**a**) λ = 1112 nm; (**b**) λ = 1196 nm; (**c**) λ = 670 nm and (**d**) λ = 673 nm. The white and black domains denote the strongest and weakest |*H*| fields, respectively.

As an example, the parameters of *θ*_dev_ = 60° and *D*_dev_ = 100 nm are chosen to simulate the *H_z_* field distributions, as shown in Figure 6. The wavelengths are set as *λ* = 1152 nm (dipole mode) and *λ* = 671 nm (quadrupole mode), which correspond to the highest transmission values of the two Fano resonances. For the symmetry-breaking case, the symmetry of the *H_z_* field distributions is destroyed, resulting in the formation of Fano resonances. Furthermore, the eigenmodes of a circular cavity with a center-deviated metal core (*θ*_dev_ = 60° and *D*_dev_ = 100 nm) are simulated, as shown in Figure 7. We can see that two eigenmodes are dipolar states and the other two are quadrupolar states. In addition, comparing Figure 2b,c and Figure 6a,b, it shows that great modification of field distributions is produced when the metal core is deviated. Thus, the coupling between the cavity (with its off-centered inclusion) and the waveguides is correspondingly changed. At some specific wavelengths, such field patterns with weak or zero field-intensity distribution at the coupling region between the right waveguide and the cavity are generated, resulting low or zero transmission at the specific wavelengths. At other wavelengths, the field distribution at the coupling region is strong, and one can have high transmission. This results in the Fano resonance phenomenon. Therefore, Fano resonances can be considered to be results induced by the symmetry breaking or geometric effect that affects the field distribution intensity at the coupling region between the right waveguide and the cavity. Such a field-distribution pattern change can be regarded as being caused by the interference between the waveguide modes and the cavity modes.

**Figure 8:**
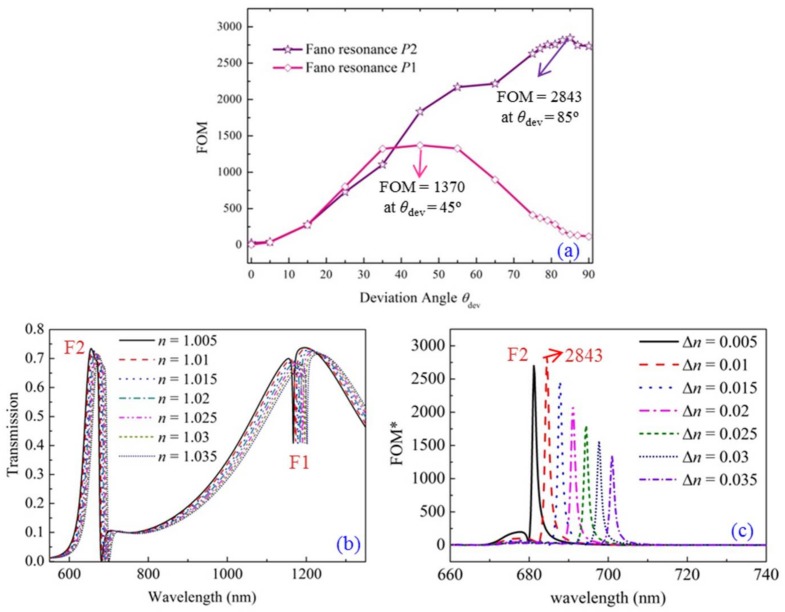
(**a**) The changes of the FOM value (Fano resonances F1 and F2) related to the parameter of deviation angle *θ*_dev_, with *D*_dev_ = 100 nm remains unchanged; (**b**) The transmissions for different background refractive index *n*; (**c**) The values FOM* = Δ*T*/(*T*Δ*n*) calculated for different background refractive index change Δ*n*.

As we know, designing sensors is one of the most important applications for Fano resonance. In order to investigate the sensitivity characteristic of Fano resonance to the refractive index of the dielectric material in structure, the figure of merit (FOM*) value is investigated. FOM* is defined as Δ*T*/(*T*Δ*n*), where *T* denotes the transmission in the proposed structure. It describes the relative transmission variation Δ*T*/*T* at a fixed wavelength induced by the refractive index change Δ*n* of the dielectric material. Letting FOM = Max(FOM*), we calculate the FOM values of Fano resonances F1 and F2 when the parameter of deviation angle *θ*_dev_ is scanned from 0° to 90°, as shown in Figure 8a. Here *D*_dev_ = 100 nm remains unchanged. At first, the FOM value becomes larger with the increase of *θ*_dev_. Then it reaches a maximum value. Afterward, the FOM value decreases with the increase of *θ*_dev_. The highest FOM values for Fano resonances F1 and F2 are 1370 (*θ*_dev_ = 45°) and 2843 (*θ*_dev_ = 85°), respectively. We think the reduction of FOM is mainly due to the fact that the transmission variation Δ*T* becomes relatively smaller. Here, we choose *θ*_dev_ = 85° as an example to show the calculation of FOM in detail, which is the maximum FOM for Fano resonance F2. Figure 8b gives the transmission related to the refractive index *n* of the dielectric material. The interval of index variation is 0.005. The Fano resonances exhibit an obvious red shift with the increase of refractive index. As a result, the designed structure is very sensitive to the change of background refractive index. The values of refractive index sensitivity of Fano resonances F1 and F2 are *δλ*/*δn* = 348 nm/RIU and 671 nm/RIU, respectively. Furthermore, Figure 8c shows the calculated FOM* at different interval of refractive index (Δ*n*). The FOM* reaches the highest value of 2843 at λ = 684 nm when Δ*n* = 0.01. The corresponding parameters are *R*_cavity_ = 260 nm, *R*_core_ = 90 nm, *θ*_dev_ = 85°, *D*_dev_ = 100 nm. The high FOM contributes to the sharp Fano lineshape at this wavelength. For laboratory experiments, the refractive indices of some materials can be tested, such as acetone, alcohol, aether and nutrition liquid. On the other hand, the system in our paper has a simple and compact structure that is appropriate for integration with other on-chip devices. This plays an important role in nano-integrated plasmonic systems.

**Figure 9:**
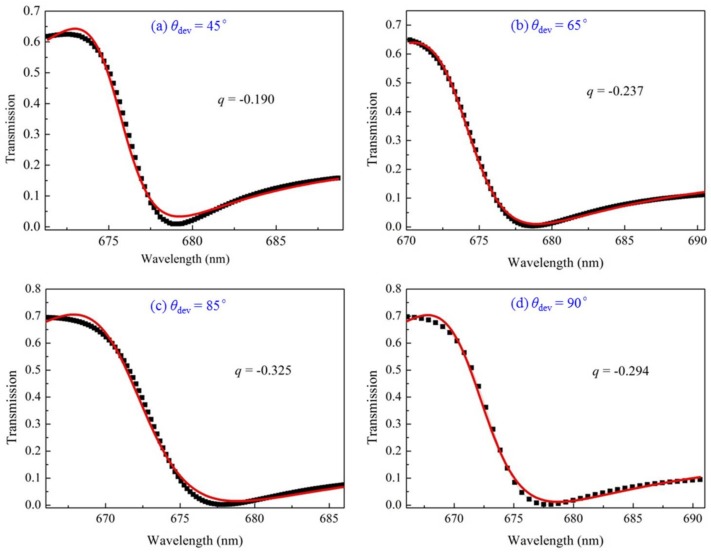
The transmissions for *θ*_dev_ = 45°, 65°, 85° and 90° (*D*_dev_ = 100 nm) are fitted by the Fano resonance formula. The black squares denote the transmission scatters and the red lines denote the fitted lines.

In order to further understand the characteristics of Fano resonances, the transmission can be expressed by the following formula [43,44],

(7) $$T=T_{Bethe}(\lambda )+C\frac{\left(\lambda -\lambda_{R\mathrm{es}}+q\Gamma /2\right)}{{\left(\lambda -\lambda_{R\mathrm{es}}\right)}^{2}+{\left(\Gamma /2\right)}^{2}}$$

where *T_Bethe_*(*λ*) is the direct transmission or referred to as Bethe’s contribution, *C* is the non-resonant transmission coefficient, *λ_Res_* is the resonant wavelength, *q* is a dimensionless parameter that describes the asymmetry profile, and *Γ* is the linewidth. Here, we focus on the Fano resonance F2. The transmissions for different deviation angles *θ*_dev_ = 45°, 65°, 85° and 90° (*D*_dev_ = 100 nm) are fitted by Fano resonance formula, as shown in Figure 9. The black squares denote the transmission scatters and the red lines denote the fitted lines. This shows that |*q*| reaches its maximum values at *θ*_dev_ = 85°, meaning that the asymmetry is high for this case.

## 4. Conclusions

In summary, we proposed a type of tunable nanosensor based on Fano resonance, which is realized in a MIM cavity system with a center-deviated metal core and two side-coupled waveguides. By changing the deviation direction and distance of the metal core in the cavity, Fano resonances can be produced and tuned. The Fano resonances can be considered to be results induced by the symmetry breaking or geometric effect that affects the field distribution intensity at the coupling region between the right waveguide and the cavity. Such a field-distribution pattern change can be regarded as being caused by the interference between the waveguide modes and the cavity modes. The investigated results show that the spectral positions and modulation depths of Fano resonances are greatly sensitive to the deviation parameters. This provides an effective method to flexibly tune Fano resonances. The proposed sensor has the advantages of compact structure, high transmission, sharp asymmetric lineshape, and high sensitivity to the background refractive index. This work may provide a useful reference for designing on-chip plasmonic nanosensors and other relevant devices.
